# Ethical requirements of instructions for authors of complementary and alternative medicine journals: a cross-sectional study

**DOI:** 10.1186/s12910-024-01077-1

**Published:** 2024-07-13

**Authors:** Chenyu Ren, Yixuan Li, Peipei Du, Xuebin Zhang, Wanjun Xue, Chi Zhang

**Affiliations:** 1 Dongzhimen Hospital, Beijing University of Chinese Medicine, 5 Haiyuncang Street, Dongcheng District, Beijing, 100070 China; 2 Institute for Brain Disorders, Beijing University of Chinese Medicine, Beijing, China

**Keywords:** Complementary and alternative medicine, Integrative and complementary medicine, Medical ethics, Instructions for authors

## Abstract

**Background:**

Medical research in complementary and alternative medicine (CAM) has increased recently, raising ethical concerns about the moral status of CAM. Medical academic journals are responsible for conducting ethical review (ER) of manuscripts to protect the interests of human subjects and to make ethical results available before deciding to publish. However, there has been no systematic analysis of the ER in CAM journals. This study is aim to evaluate the current status of ethical requirements and compliance in CAM journals.

**Methods:**

This is a cross-sectional study. We reviewed instructions for authors (IFAs) of CAM journals included in the Journal Citation Reports (2021) (https://jcr.clarivate.com) for general information and requirements for ER. We also browsed the manuscripts regarding randomized controlled trials published by CAM journals in Q1 and Q2 section from January to June, 2023, to check the actual situation of ethical requirement. Descriptive statistics and Fisher’s exact test were used for statistical analysis.

**Results:**

27 journals and 68 manuscripts were ultimately included. 92.6% (25/27) IFAs included keywords of ER, indicating the presence of ethical considerations. However, no specific ER was required for CAM (*n* = 0). We categorized journals by Geographic origin, JCR section, Year of electronic JCR, Types of studies, % of OA Gold to explore the factors that could influence CAM journals to have certain ethical review policies. The results showed there was no statistical significance in certain ethical review policy in any classification of journals (*p* > 0.05). All RCT manuscripts included in the study generally met the requirements of the published journals for ethical review.

**Conclusions:**

All IFAs discussed ER, but the content was scattered, unfocused, and there were no specific ER requirements regarding CAM. Although the manuscripts basically met the requirements of the journal, it was not possible to get closer to the process of ER in the manuscript. To ensure full implementation of these policies in the future, CAM journals should require authors to provide more details, or to form a list of items necessary for CAM ethical review.

## Introduction

Medical research propels medical advancement. Following the completion of numerous non-clinical studies, clinical trials in human subjects must be conducted to conclusively validate or reveal the efficacy and safety of a putative intervention in humans. Nearly 300,000 patients taken part in clinical trials from 2015 to 2019 [1]. According to the World Health Organization (WHO) report [2], the number of newly recruited trials registered on the International Clinical Trials Registry Platform (ICTRP) increased steadily in most WHO regions from 1999 to 2022. On 29th May, 2023, over 453,000 studies from 221 countries were registered on ClinicalTrials.gov [3]. Such clinical trials were likely to pose potential risks, even fatal injuries, to people who took part in them. Therefore, strict adherence to the ethical code and the protection of the rights, interests, health and safety of trial subjects are primary principles that cannot be ignored in clinical trials. For this reason, four of the world’s most important and universal guiding principles, namely the Nuremberg Code [4], the Declaration of Helsinki [5], the Belmont Report [6] and the International Ethical Guidelines for Health-related Research Involving Humans [7] concerning human subjects have been formulated in the world to protect human subjects in medical research.

As a type of media, a medical academic journal is a platform for the dissemination of information about medical research. Publication in peer-reviewed journals (PRJs) is still the most fundamental way medical research is disseminated, and PRJs are expected to publish accurate information, share knowledge and advance scientific research [8–10]. In order to protect the interests of human subjects in clinical trials and to make ethical trial results available for publication, medical journals have a responsibility and obligation to conduct a rigorous ethical review of manuscripts when deciding whether to publish them, i.e. “no ethics, no publication”. Guidelines such as the International Committee of Medical Journal Editors’ (ICMJE) Recommendations for the Conduct, Reporting, Editing, and Publication of Scholarly Work in Medical Journals (previously known as Uniform Requirements for Manuscripts) [11] and the Committee on Publication Ethics’ (COPE) Core Practice [12] also require ethical review of authors when writing medical research manuscripts.

Complementary and alternative medicine (CAM) is defined as an alternative to mainstream medicine that can complement the deficiencies of mainstream medicine and provide methods of diagnosis, treatment and prevention that cannot be achieved by mainstream medicine [13]. CAM is widely used around the world. International prevalence estimates vary widely, ranging from 10% to 76% [14]. According to statistics of WHO regional offices [15, 16], more than 100 million people in Europe use CAM, and one fifth of them use CAM on a regular basis. CAM is more widely used in Asia, Africa, Australia and North America. With the popularity of CAM therapies, more and more related medical researches have emerged, raising fundamental ethical questions about the moral status of CAM [17].

However, it is unclear whether the ethical review process is rigorous, and whether the various CAM journals that publish the results of clinical trials are doing so. There have been studies on the status of ethical review in medical journals, but there is no systematic research on the content of ethical review in CAM journals. The aim of this study is to assess the current state of ethical requirements in CAM journals by analyzing the instructions for authors (IFAs) of CAM journals, meanwhile, we also want to check the actual situation of the ethical requirement of manuscripts published by these CAM journals.

## Methods

### Data source

We conducted a cross-sectional study of instructions, guides and other comparable documents for authors of journals in the integrative & alternative medicine category of 2021 Journal Citation Reports (JCR). All official websites were browsed, and every IFA or other similar texts of each journal were downloaded between April to May 2023. We also browsed the manuscripts regarding randomized controlled trials published by CAM journals in Q1 and Q2 section from January to June, 2023, to check the actual situation of ethical requirement.

### Selection criteria

**Fig. 1:**
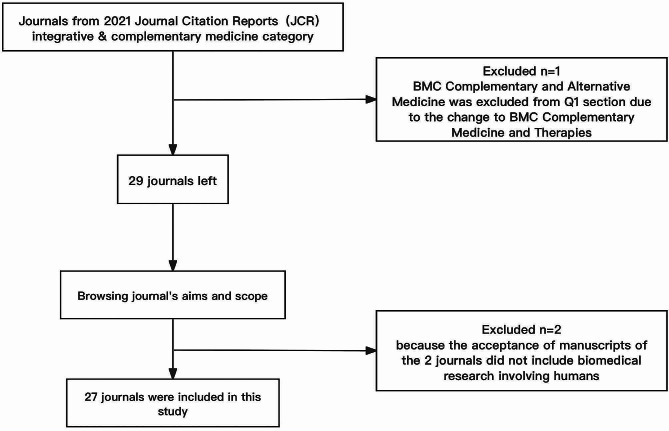
Study flow diagram

Journals met the following criteria: (1) be classified as CAM in JCR (2021); (2) with a scope of medical research involving human subjects, were included (Fig. 1).

Manuscripts met the following criteria: (1) published by CAM journals in Q1 and Q2 section from January to June, 2023; (2) the type of study was randomized controlled trials, were included.

### Data extraction

According to predefined selection criteria, two authors (CYR, YXL) were independent to each other to browse the official website of JCR (https://jcr.clarivate.com) to obtain the list of CAM journals, after screening the “aims & scope” of each journal on their websites, the journals did not fulfill the inclusion criteria were removed. The full texts of the remaining IFAs of journals were independently screened by the two authors above. Two authors (CYR, YXL) adopted a predefined data extraction form to collect information of IFAs for this study. The data extraction form consists of the following contents: (1) general information (names, regions/countries, publishers, impact factor of 2021, JCR sections, years of electronic JCR, % OA GOLD, etc.); (2) whether the keywords “ethic(s)”, “ethical”, or “human” were contained in the subtitles or text words of IFAs; (3) whether an statement of ethical review approval from institutional review board (IRB) was required in the manuscript; (4) whether the IRB approval number was required in the manuscript; (5) whether the name of IRB was required in the manuscript; (6) citation situation of the Declaration of Helsinki (DoH), ICMJE Recommendations, and COPE Core Practice; (7) whether registration was required and whether the research was conducted according to reporting guidelines; (8) policies of informed consent (IC), images privacy and data sharing.

As for the extracted data from manuscripts, three authors (CYR, PPD and XBZ) reviewed the titles and abstracts of the manuscripts published by CAM journals in Q1 and Q2 section on their official websites from January to June, 2023, independently. The articles that did not fulfill the inclusion criteria were removed. The full texts of the remaining articles were independently screened by the two authors (CYR, PPD), using a predefined data extraction form that collected information for this study. Contents of the form included: (1) registration information (including registration website and number); (2) name of IRB and IRB approval number; (3) whether handwritten IC was obtained; (4) citation situation of the DoH, ICMJE Recommendations, and COPE Core Practice. Two authors above (CYR, PPD) extracted the data from each trial record independently.

In the data extraction process described above, any disagreements in primary and full-text screening were discussed to be resolved. A third author (CZ) was consulted if needed.

### Statistical analysis

Results were presented as frequencies and percentages for categorical data. Differences between groups were tested using the Fisher’s exact test, with a significance level of *p* < 0.05. We used Excel software to enter all data, and statistical analysis was performed using IBM SPSS version 26 (IBM, Armonk, NY, USA).

### Classifications of journals

We categorized journals by Geographic origin, JCR section, Year of electronic JCR, Types of studies, % of OA Gold to explore the factors that could influence CAM journals to have certain ethical review policies. In terms of geographical origin, we classified journals into two groups, European and American area, and Asia. We also grouped journals according to JCR sections, with journals from Q1 section and journals from Q2-Q4. We counted the years until 2021 when CAM journals were first included in the JCR, under this classification, we grouped journals into those with < 11.93 years and ≥ 11.93 years. We determined the cutoff point by using the mean value because the Shapiro–Wilk (S-W) test showed that the stata followed a normal distribution (*p* = 0.121). % of OA Gold was counted as well, we determined the cutoff point by using the median (< 8.99% or ≥ 8.99%) because the S–W test showed that the stata did not followed a normal distribution (*p* < 0.001). Additionally, we classified journals according to the types of studies. Most journals required original research for submission, but some journals specifically offered to receive clinical trials, RCTs, case reports, protocol, pilot study, etc. We also divided these journals into two categories.

## Results

Initially, 30 CAM journals were retrieved from JCR (2021). After reviewing the aims and scope of each journal, PLANTA MEDICA and Boletín Latinoamericano y del Caribe de Plantas Medicinales y Aromáticas were excluded because the acceptance scopes of these 2 journals did not include biomedical research involving humans, BMC Complementary and Alternative Medicine was excluded from Q1 section due to the change to BMC Complementary Medicine and Therapies, finally, 27 journals remained.

### General information about included journals

**Fig. 2:**
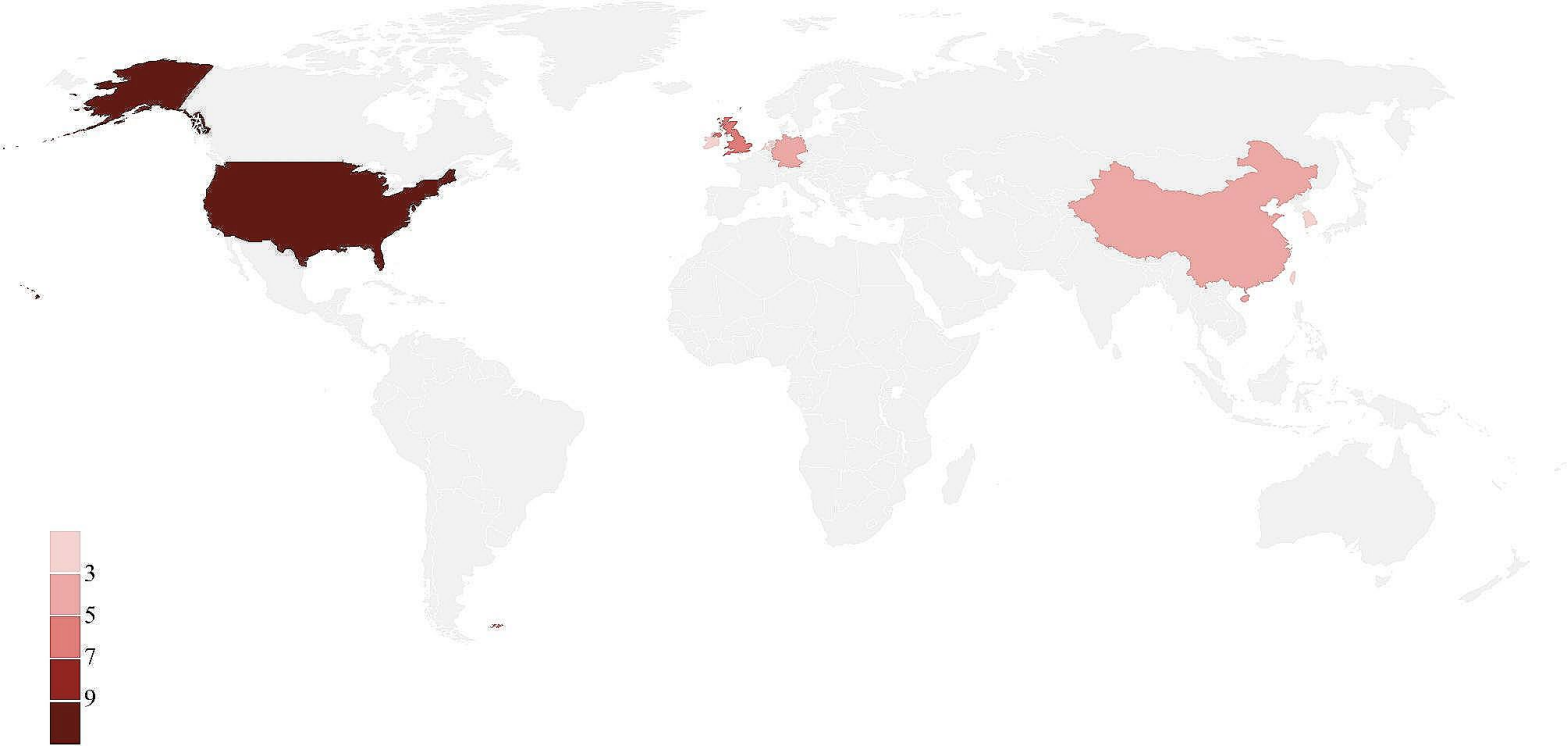
Countries and regions of 27 CAM journals

The 27 journals originated in eight different nations and areas (Table 1). Ten journals were from the United States, five from England, four from China’s mainland, three from Germany, two from South Korea, and one each from Ireland, the Netherlands, and Taiwan, China (Fig. 2). 27 journals were published by 12 different publishers.

**Table 1:** General information about included journals

Name	Countries /Regions	2021 IF	JCR	Years of electronic JCR	% of OA Gold	DoH	ICMJE	COPE	IRB approval	IRB number	IRB name	Registration	Reporting guidelines	IC	Images privacy	Data sharing
Phytomedicine	Germany	6.656	Q1	23	8.99%	√		√					√	√		√
American Journal of Chinese Medicine	United States	6.005	Q1	24	1.74%	√	√	√		√	√	√	√	√		√
Journal of Ginseng Research	South Korea	5.735	Q1	9	99.6%	√	√	√						√		√
Journal of ETHNOPHARMACOLOGY	Ireland	5.195	Q1	24	5.42%	√		√						√		√
Chinese Medicine	England	4.546	Q1	8	100%	√	√	√	√	√	√	√	√	√	√	√
Integrative Medicine Research	South Korea	4.473	Q1	2	99.44%	√	√	√	√	√	√	√	√	√	√	√
Journal of Traditional and Complementary Medicine	Taiwan, China	4.221	Q2	0	98.95%	√	√	√	√					√	√	√
Journal of Integrative Medicine-JIM	China	3.951	Q2	2	7.07%			√								√
Chinese Journal of Natural Medicines	China	3.887	Q2	7	0.00%			√								√
Complementary Therapies in Clinical Practice	Netherlands	3.577	Q2	6	6.99%	√	√	√				√	√			√
COMPLEMENTARY THERAPIES IN MEDICINE	England	3.335	Q2	19	27.68%	√	√	√	√			√	√	√	√	√
INTEGRATIVE CANCER THERAPIES	United States	3.077	Q2	13	98.71%	√	√	√	√	√	√	√	√	√		√
BMC Complementary Medicine and Therapies	England	2.838	Q2	1	99.25%	√	√	√	√	√	√	√	√	√	√	√
Evidence-based Complementary and Alternative Medicine	England	2.65	Q3	14	98.58%	√		√	√			√	√	√		√
Chinese Journal of Integrative Medicine	China	2.626	Q3	12	0.00%		√	√	√			√	√	√	√	
Journal of Traditional Chinese Medicine	China	2.547	Q3	10	0.00%	√		√	√		√	√	√	√		
Journal of Herbal Medicine	England	2.542	Q3	8	1.52%	√	√	√	√			√	√	√	√	√
JOURNAL OF ALTERNATIVE AND COMPLEMENTARY MEDICINE	United States	2.381	Q3	8	9.57%	√	√	√	√		√	√	√	√	√	√
Explore-The Journal of Science and Healing	United States	2.358	Q3	16	14.29%	√	√		√		√			√	√	√
Acupuncture in Medicine	United States	1.976	Q3	11	8.70%	√	√	√	√	√	√	√	√	√	√	√
Homeopathy	United States	1.818	Q4	13	8.26%	√	√	√				√	√	√	√	
European Journal of Integrative Medicine	Germany	1.813	Q4	11	10.58%	√	√	√	√			√	√	√	√	√
ALTERNATIVE THERAPIES IN HEALTH AND MEDICINE	United States	1.804	Q4	21	0.00%	√	√		√			√	√	√	√	
Complementary Medicine Research	Germany	1.449	Q4	4	14.97%	√	√	√	√	√	√	√	√	√		√
JOURNAL OF MANIPULATIVE AND PHYSIOLOGICAL THERAPEUTICS	United States	1.3	Q4	21	0.90%	√	√	√	√			√	√	√	√	√
Holistic Nursing Practice	United States	1.226	Q4	11	0.77%											
ACUPUNCTURE & ELECTRO-THERAPEUTICS RESEARCH	United States	0.684	Q4	24	13.64%			√	√							

JCR: Journal Citation Reports; DoH: the Declaration of Helsinki; ICMJE: International Committee of Medical Journal Editors; COPE: Committee on Publication Ethics; IRB: Institutional Review Board; IC: Informed Consent

### The requirements for ethical review in IFAs

Of the 27 journals included in the study, 92.6% (25/27) IFAs contained keywords of “ethic(s)”, “ethical”, or “human” in the subtitles and text words, which represented there were ethical considerations in these journals. Of these, 84.0% (21/25) explicitly mentioned that the manuscript of biomedical research involving human subjects should undergo ethical review; 12.0% (3/25) IFAs’ (Journal of Integrative Medicine, Journal of Herbal Medicine, ACUPUNCTURE & ELECTRO-THERAPEUTICS RESEARCH) content of ethical review regarding the policy of publishing ethics most and authors needed to read IFAs carefully to search for key information; 4.0% (1/25) only mentioned ethics in publication (Holistic Nursing Practice). 7.4% (2/27) IFAs of journals (Phytomedicine, Chinese Journal of Integrative Medicine) had no clear claim to ethical review of medical research involving human subjects in the subtitles or text words of their IFAs, but there was an “ethics disclosures” on the official website page of Chinese Journal of Integrative Medicine, IFA of Phytomedicine declared that “the author should ensure that the manuscript contains a statement that all procedures were performed in compliance with relevant laws and institutional guidelines and that the appropriate institutional committee(s) have approved them”.

### Citation situation of the DoH, ICMJE recommendations, and COPE Core Practice

In this study, 81.5% (22/27) of the IFAs mentioned the Declaration of Helsinki (DoH), 70.4% (19/27) of the IFAs mentioned ICMJE recommendations. 21 journals are members of COPE, although 3 journals had not yet become members of COPE, their IFAs also required authors to follow COPE core practice.

In addition to the above international general guidelines for ethical review, the IFAs of Phytomedicine recommended that authors comply with ICH-E6 Good Clinical Practice [17]. Policy Statement on Geopolitical Intrusion on Editorial Decisions issued by the World Association of Medical Editors (WAME), UK’s The Medicines for Human Use (Clinical Trials) Regulations, ICMJE Recommendations for the Protection of Research Participants, NLM’s Research Reporting Guidelines, and Initiatives and Guidelines for the Conduct of Human Embryonic Stem Cell Research established by the International Society for Stem Cell Research (ISSCR) always appeared in IFAs.

The results showed there was no statistical significance in the citation of the DoH, ICMJE recommendations and COPE core practice in any classification of journals (*p* > 0.05, Table 2). For the factors related to journals that we have taken into account above, they were not influencing factors for CAM journals to make a particular ethical review request. We integrated journals that cited the above three documents (the DoH, ICMJE recommendations, COPE guidelines) at the same time, and calculated their mean value IF: 3.20 (3.05), mean value years of electronic JCR: 8.8 (14.9), median % OA GOLD: 14.97% (5.42%), and the data of journals that do not cite the above three documents are in parentheses.

**Table 2:** Content of ethical review of journals of Q1 section versus journals of Q2-Q4 section

Content	Geographic origin				JCR Quartile rankings			Year of electronic JCR			Types of studies			% of OA Gold		
	Total	European and American area	Asia	*P* value*	Q1	Q2-Q4	*P* value*	< 11.93	≥ 11.93	*P* value*	original research	additional key words^#^	*P* value*	< 8.99%	≥ 8.99%	*P* value*
**Declaration of Helsinki (%)**																
yes	22(81.5)	18(81.8)	4(18.2)	0.091	6(27.3)	16(72.7)	0.555	12(54.5)	10(45.5)	1.000	10(45.5)	12(54.5)	0.618	10(45.5)	12(54.5)	0.326
no	5(18.5)	2(40.0)	3(60.0)		0(0.0)	5(100.0)		3(60.0)	2(40.0)		1(20.0)	4(80.0)		4(80.0)	1(20.0)	
**ICMJE Recommendations (%)**																
yes	19(70.4)	15(78.9)	4(21.1)	0.633	4(21.1)	15(78.9)	1.000	11(57.9)	8(42.1)	1.000	7(36.8)	12(63.2)	0.675	9(47.4)	10(52.6)	0.678
no	8(29.6)	5(62.5)	3(37.5)		2(25.0)	6(75.0)		4(50.0)	4(50.0)		4(50.0)	4(50.0)		5(62.5)	3(37.5)	
**COPE core practice (%)**																
yes	24(88.9)	17(70.8)	7(29.2)	0.545	6(25.0)	18(75.0)	1.000	14(58.3)	10(41.7)	0.569	11(45.8)	13(54.2)	0.248	12(50.0)	12(50.0)	1.000
no	3(11.1)	3(100.0)	0(0.0)		0(0.0)	3(100.0)		1(33.3)	2(66.7)		0(0.0)	3(100.0)		2(66.7)	1(33.3)	
**IRB approval(%)**																
yes	18(66.7)	14(77.8)	4(22.2)	0.653	2(11.1)	16(88.9)	0.136	10(55.6)	8(44.4)	1.000	6(33.3)	12(66.7)	0.411	7(38.9)	11(61.1)	0.103
no	9(33.3)	6(66.7)	3(33.3)		4(44.4)	5(55.6)		5(55.6)	4(44.4)		5(55.6)	4(44.4)		7(38.9)	2(22.2)	
**IRB number(%)**																
yes	7(25.9)	6(85.7)	1(14.3)	0.633	3(42.9)	4(57.1)	0.290	5(71.4)	2(28.6)	0.408	5(71.4)	2(28.6)	0.084	2(28.6)	5(71.4)	0.209
no	20(74.1)	14(70.0)	6(30.0)		3(15.0)	17(85.0)		10(50.0)	10(50.0)		6(30.0)	14(70)		12(60.0)	8(40.0)	
**IRB name(%)**																
yes	10(37.0)	8(80.0)	2(20.0)	0.678	3(30.0)	7(70.0)	0.638	7(70.0)	3(30.0)	0.424	6(60.0)	4(40.0)	0.224	4(40.0)	6(60.0)	0.440
no	17(63.0)	12(70.6)	5(29.4)		3(17.6)	14(82.4)		8(47.1)	9(52.9)		5(29.4)	12(70.6)		10(58.8)	7(41.2)	
**Registration (%)**																
yes	18(66.7)	15(83.3)	3(16.7)	0.175	3(16.7)	15(83.3)	0.367	10(55.6)	8(44.4)	1.000	6(33.3)	12(66.7)	0.411	10(55.6)	8(44.4)	0.695
no	9(33.3)	5(55.6)	4(44.4)		3(33.3)	6(66.7)		5(55.6)	4(44.4)		5(55.6)	4(44.4)		4(44.4)	5(55.6)	
**Reporting guidelines (%)**																
yes	19(70.4)	16(84.2)	3(15.8)	0.145	4(21.1)	15(78.9)	1.000	10(52.6)	9(47.4)	0.696	7(36.8)	12(63.2)	0.675	10(52.6)	9(47.4)	1.000
no	8(29.6)	4(50.0)	4(50.0)		2(25.0)	6(75.0)		5(62.5)	3(37.5)		4(50.0)	4(50.0)		4(50.0)	4(50.0)	
**Informed consent(%)**																
yes	22(81.5)	17(77.3)	5(22.7)	0.580	6(27.3)	16(72.7)	0.555	11(50.0)	11(50.0)	0.342	10(45.5)	12(54.5)	0.618	10(45.5)	12(54.5)	0.326
no	5(18.5)	3(60.0)	2(40.0)		0(0.0)	5(100.0)		4(80.0)	1(20.0)		1(20.0)	4(80.0)		4(80.0)	1(20.0)	
**Images policy(%)**																
yes	14(51.9)	11(78.6)	3(21.4)	0.678	2(14.3)	12(85.7)	0.385	8(57.1)	6(42.9)	1.000	4(28.6)	10(71.4)	0.252	7(50.0)	7(50.0)	1.000
no	13(48.1)	9(69.2)	4(30.8)		4(30.8)	9(69.2)		7(53.8)	6(46.2)		7(53.8)	6(46.2)		7(53.8)	6(46.2)	
**Data sharing(%)**																
yes	21(77.8)	16(76.2)	5(23.8)	0.633	6(28.6)	15(71.4)	0.284	13(6.9)	8(38.1)	0.357	10(47.6)	11(52.4)	0.350	9(42.9)	12(57.1)	0.165
no	6(22.2)	4(66.7)	2(33.3)		0(0.0)	6(100.0)		2(33.3)	4(66.7)		1(16.7)	5(83.3)		5(83.3)	1(16.7)	

*Fisher’s exact test

^#^with key words of randomized controlled trials, case report, protocol, pilot study, clinical trials, etc

### The requirements of IRB approval, name of IRB, IRB approval number, registration and reporting guidelines

Many journals also requested informations on IRB, but Geographic origin, JCR section, Year of electronic JCR, Types of studies, % of OA Gold were not associated with requirements of IRB approval, name of IRB, IRB approval number, registration and reporting guidelines separately (*p* > 0.05, Table 2). Some journals required that the authors provide the details (JOURNAL OF ALTERNATIVE AND COMPLEMENTARY MEDICINE) of the ethical review process and the date of ethical review (American Journal of Chinese Medicine) along with manuscripts.

### Policies of IC, images privacy and data sharing

In addition to adhering to the international guidelines mentioned above, some journals emphasized the principles of IC and patient privacy. 81.5% (22/27) of journals mentioned obtaining IC from patients, some of the journals (Journal of Traditional and Complementary Medicine, COMPLEMENTARY THERAPIES IN MEDICINE, INTEGRATIVE CANCER THERAPIES) required that patients’ handwritten IC be retained and backed up, some of the journals (Complementary Medicine Research, JOURNAL OF MANIPULATIVE AND PHYSIOLOGICAL THERAPEUTICS) required authors to provide a statement of detailed procedure in obtaining IC. Among these journals, 50.0% (11/22) of them proposed protecting patient privacy as well. As for the use of patients’ images and photographs, 51.9% (14/27) of the journals emphasized the need to obtain IC from patients before using their photos, and that some identifying information should be hidden, but there were no more separate and specific consent was required. 77.8% (21/27) of journals promoted data sharing and make research more transparent, these journals encouraged authors to share their research data, which refers to the “results of observations or experimentation that validate research findings”, but there were no policies relevant for personal data protection.

We integrated journals which had a requirement of IRB approval, name of IRB, IRB approval number, registration, reporting guidelines along with IC, and calculated their mean value IF: 2.96 (3.19), mean value years of electronic JCR: 6.7 (13.7), median % OA GOLD: 98.71% (7.03%), and the data of journals that do not have the requirements above are in parentheses.

It seems that CAM journals which were included in electronic JCR in recent years, and the higher the % OA GOLD, will have more comprehensive requirements for ethical review.

### The actual situation of ethical requirement in published manuscripts

We also browsed the manuscripts regarding randomized controlled trials (RCT) published by CAM journals in Q1 and Q2 section from January to June, 2023, to check the actual situation of ethical requirement. There were 68 manuscripts (20 from Q1 section, 48 from Q2 section) in total (Table 3). Of the 20 randomized controlled studies included in Q1, 11 studies were from China, 4 from Korea, 2 from Iran, and 1 from Brazil, Australia, and the United States, respectively. 95.00% (19/20) manuscripts mentioned that their research had been registered on the website, 90.00% (18/20) of which also gave registration numbers, and only one [18] did not mention any registration information about the clinical trial. Of the 48 randomized controlled studies included in Q2, 95.83% (46/48) manuscripts mentioned that their research had been registered on the website, 91.67% (44/48) of which gave registration numbers, 4.17% (2/48) manuscripts [19, 20] did not contain the information about trial registration. In Q1 section, all the manuscripts mentioned the name of the REC/IRB, and 95% (19/20) of the studies also clearly indicated the ethics review number, while one study, from China [21] published in Chinese Medicine did not specify the ethics review number, which is actually not in accordance with the requirements of the journal. In Q2 section, 6.25% (3/48) manuscripts did not mention any information of REC/IRB and ethics review number. Of the remaining 45 studies, 3 studies published in Complementary Therapies in Clinical Practice and 1 study published in COMPLEMENTARY THERAPIES IN MEDICINE only had the name of the ethics review committee, which was not requested by either journal, and 2 studies published in INTEGRATIVE CANCER THERAPIES and 3 studies published in BMC Complementary Medicine and Therapies only had the name of the ethics review committee without mentioning the ethics review number either, although both journals made clear requests for the provision of the ethics review number.

**Table 3:** Actual situation of ethical requirement in published manuscripts in Q1 and Q2 section

	Q1 (*n* = 20)	Q2 (*n* = 48)	total (*n* = 68)
Registration (%)	19 (95.00)	46 (95.83)	65 (95.56)
Registration number (%)	18 (90.00)	44 (91.67)	62 (91.18)
IRB name	20 (100.00)	9 (18.75)	29 (42.65)
IRB number	19 (95.00)	45(93.75)	64 (94.18)
IC	19 (95.00)	35 (72.92)	54 (79.41)
DoH	4 (20.00)	28 (58.33)	32 (47.06)
ICMJE recommendations	0 (0.00)	0 (0.00)	0 (0.00)
COPE core practice	0 (0.00)	0 (0.00)	0 (0.00)

DoH: the Declaration of Helsinki; ICMJE: International Committee of Medical Journal Editors; COPE: Committee on Publication Ethics; IRB: Institutional Review Board; IC: Informed Consent

As for obtaining IC forms from patients, it was obtained from study subjects in all 68 studies, 95% (19/20) studies in Q1 section and 72.92% (35/48) studies in Q2 section mentioned that it was signing an IC form, the others were unknown.

Of all 68 manuscripts, only 4 manuscripts in Q1 section mentioned compliance with the DoH, while 28 manuscripts in Q2 section mentioned the DoH. Beyond that, there was no reference to other internationally recognized guiding principles mentioned.

## Discussions

A total of 27 IFAs of CAM journals were included, most of which have ethical review requirements for manuscripts. However, there was no ethical review requirement specific to CAM. All RCT manuscripts included in the study also generally met the requirements of the published journals for ethical review.

The ethical review references mostly observe the DoH, ICMJE recommendations, COPE guidelines. Some journals will refer to the relevant rules of the professional field to review the format and content of manuscripts. For biomedical research involving human subjects, journals will place greater emphasis on informed consent and privacy. However, there was no ethical review requirement specific to CAM in this study, there is no standard and accepted review list to help journal editors and authors to review and address the deficiencies and the ethical review of CAM manuscripts was more about censorship of form than content.

### Ethical review of CAM manuscripts required

CAM includes numerous types of therapies, including acupuncture, chiropractic therapy, herbal therapy, homeopathy, and osteopathy [22]. Many CAM therapies are claimed by the public to be effective and safe, however, clinical evidence supporting the use of CAM products is insufficient [23]. Any intervention should be tested for efficacy and safety by completing clinical trials before it can be applied in real clinical settings, and ethical review before clinical trials is essential. In recent years, important ethical debates about the moral standing of CAM have been sparked by the growth of CAM medical research.

Ethical review, which we are now doing to protect the safety of our subjects, should be done whenever people are involved as volunteers [24]. In our study, the vast majority of CAM journals in the JCR (2021) contain ethical review requirements for biomedical research involving humans, and RCT published in these journals generally meet the requirements of the respective journals. We found that CAM journals required authors to simply declare that “the study was approved by the IRB” in Abstract or Methods section and did not require further details of the ethical review process.

As a type of media for publishing academic results, medical journals have the responsibility and obligation to conduct ethical review of the manuscripts involved, but such ethical review should not be a mere formality. The current situation is that although most journals require investigators to provide medical research registration numbers, ethics committee names, and ethics review numbers, and also mention in the text that the research complies with the DoH, GCP, and other internationally recognized programs for the protection of human subjects in clinical research, it is sometimes difficult to determine whether the investigator is providing the appropriate information to comply with publication requirements, and it is not possible to go further to determine the more specific content of the ethical review of a particular study. We also hope that some supporting materials can prove that all the ER processes are serious, rigorous and ethical.

### Obtain informed consent in general and special populations

Informed consent (IC) is a universally accepted principle in modern society, an integral and imperative component of medical practice, and is at the core of the protection of human subjects in clinical trials. It is the process by which clinicians communicate with patients to help them better understand the significant risks and benefits of diagnostic or treatment alternatives, including not taking any measures that affect their health and disease status [25].

Obtaining IC from research subjects in CAM research might be more complex than in conventional biomedical research because it involves multiple modalities, it is usually provided by non-MD practitioners who might differ greatly one from one another, and uses a language that is not always clear to patients [26].

However, the reality was that the studies published in the journals only briefly mention that the study obtained IC from the research subjects, but there was no detailed description of how the process of obtaining fully informed and voluntary consent was carried out. The process of obtaining IC for special populations, such as premature infants, infants and young children, pregnant women, and patients with cognitive disorders, was of even greater concern. It is worth mentioning that a study from China [27], which verified the efficacy and safety of Chinese herbal medicine children’s Zibei Xuanfei syrup in treating acute trachea-bronchitis with wind-heat invading lung syndrome, described in detail how to obtain informed consent from young children.

According to the American Medical Association, the core of IC is “the patient’s right of self-decision (which) can be effectively exercised only if the patient possesses enough information to enable an informed choice” [28]. So it is time to adopt a standard fully informed decision making standard for all health care practices that emphasizes meaningful dialogue between providers and patients, rather than selective, one-way, and obligatory disclosure of alternatives, risks, and benefits by providers [25].

### Ethical consideration of placebo application in CAM

The biggest problem with the use of placebos in clinical research is ethical issue [29]. In clinical trials of new drugs, it is reasonable and necessary to set up placebo control group in order to evaluate the efficacy and safety of experimental drugs objectively and accurately. Placebo controls have a range of settings and scenarios, it is important to note that placebos are not available in all new drug trials. Placebos are not usually used as a control in patients who are acutely or seriously ill or who have more severe organic lesions, nor are they generally appropriate when an existing treatment is known to prevent progression of the subject’s disease, which is concerned to be non-ethical. When a new drug is being tested in a clinical trial for a disease for which there is no known effective drug, there are usually no ethical concerns about conducting a comparative trial between the new drug and a placebo. In this point, placebo-controlled trials of any new drug or intervention in CAM should be subject to the same high standards of ethical review as mainstream medicine, TIDieR-Placebo checklist [30] assists the reporting of adequate descriptions of placebo. But the reality is the definition and composition of placebo might not be clear in the CAM clinical trials, and participates may not be fully informed to some extent. Results of a cross-sectional study regarding placebo for trials of herbal medicine treatment in rare diseases showed that there were about half of the trials (27/55, 49.1%) did not provide ethical approvals, and only one trial had details of informed consent. None of the studies were fully reported and more than half of the items reported less than 50% [31]. Taking traditional Chinese medicine as an example, the ingredients of herbal medicine are complex, and which is especially true of Chinese medicine compounding. Furthermore, the technology and process of preparing placebos may be different from that of chemical drugs. We believe that the list of CAM-related placebo reporting should be expanded based on TIDieR to regulate the preparation of placebos and make it meet the requirements of ethical review.

### Actively promote registration of trials and complete it according to the reporting guidelines

In September 2004, ICMJE released a joint statement requiring registration of all clinically directive trials prior to enrollment of the first patient for a trial to be considered for publication by medical journals that are members of the ICMJE [32]. One year later, in 2005, the ICMJE expanded the initial statement by including a requirement that a mandated deposition of the detailed information about the trial design be included in the designated trial depository [33]. This requirement has been adopted by most major medical journals worldwide that subscribe to ICMJE policies, including journals in CAM. Mandatory clinical trials registration has a critical potential impact on the eligibility of a study for editorial review and publication. It is therefore imperative for authors to be familiar and compliant with this policy prior to initiating a clinical trial.

The International Traditional Medicine Clinical Trial Registry (ITMCTR, http://itmctr.ccebtcm.org.cn/) officially joined the World Health Organization’s International Clinical Trial Registry on February 22, 2023. Become an international first-class clinical trial registration platform. ITMCTR’s primary responsibility is to accept clinical trial registrations for traditional medicine worldwide, including traditional Chinese medicine, acupuncture, massage, herbal medicine, Ayurveda, homeopathy, Unani medicine, complementary and alternative medicine. At the same time, ITMCTR will actively cooperate with all kinds of traditional medicine around the world, gradually promote the global consensus of traditional medicine clinical research norms, and improve the quality of traditional medicine evidence.

### Ethical review checklist may be the future

There is no evidence that better instructions for authors produce better articles [34]. However, publication guidelines such as Consolidated Standards for Reporting of Trials (CONSORT) are designed to improve the quality of medical research, there is some evidence that they work [35]. Checklist of ethical review in CAM journals may be formed to normative it. But the content of it should be demonstrated.

### Strengths and limitations

There are few studies that systematically summarize the ethical review of manuscripts in CAM journals, which is our strength. Our study had certain limitations. Although we included all journals from JCR (2021) CAM category, but there are still CAM journals left uncounted on other bills such as SCImago Journal Rank (SJR). We also want to increase cross-cutting comparisons with other types of journals. Furthermore, it is better to analyse the practices of ethic review of all published manuscripts.

## Conclusions

At present, most of CAM journals have ER requirements for manuscripts. The references of ER mostly observed international general guidelines, but the content was scattered, unfocused, and there were no specific ER requirements regarding CAM. Although the manuscripts basically met the requirements of the journal, it is not possible to get closer to the process of ER in the manuscript. Furthermore, there was no standard and accepted ethical review list to help CAM journal editors and authors to check out and make up for the deficiencies, so it is essential that CAM journals should require authors to provide more details, or to form a list of items necessary for CAM ethical reviews.

Every year, there will be an update of journals’ catalogues and categories in JCR, the journals’ impact factor and citation quartile will also change, the same goes for IFAs of journals. We are committed to monitoring these developments and conducting pertinent research in the future, in order to promote the ethical review of CAM journals.

## Data Availability

Not applicable. Data in this study did not come from a single dataset.

## Abbreviations

CAM: Complementary and Alternative Medicine
IFA(s): Instructions for Authors
ER: ethical review
JCR: Journal Citation Reports
DoH: the Declaration of Helsinki
PRJs: peer-reviewed journals
ICMJE: International Committee of Medical Journal Editors
COPE: Committee on Publication Ethics’
IRB: institutional review board
IC: informed consent
S-W: Shapiro–Wilk
